# Prevalence and Associated Risk Factors of Human Intestinal Helminths Parasitic Infections in Ethiopia: A Systematic Review and Meta-Analysis

**DOI:** 10.1155/2022/3905963

**Published:** 2022-08-31

**Authors:** Minichil Liyih, Destaw Damtie, Dires Tegen

**Affiliations:** ^1^Abune Gorgorios Schools, Bahir Dar Branch, Bahir Dar, Ethiopia; ^2^Bahir Dar University, College of Sciences, Department of Biology, Bahir Dar, Ethiopia; ^3^Dera Woreda Education Office, South Gondar Zone, Gondar, Ethiopia

## Abstract

**Background:**

Intestinal helminth infections are still public health problems in tropical and subtropical countries including Ethiopia. This review and meta-analysis aimed to produce the pooled prevalence and associated risk factors of human intestinal helminth parasitic infections (HIHPIs) in Ethiopia.

**Methods:**

Articles written in English were searched from online databases. Sixty-seven studies were included. Meta-analysis was computed using STATA version 14.

**Result:**

The pooled prevalence of HIHPIs was (33.35%, 95% CI: 28.85%, 37.86%). *Ascaris lumbricoides* (10.84%, 95% CI: 9.34, 12.34), hookworm spp. (8.89%, 95% CI: 7.75, 10.04), *Schistosoma mansoni* (4.22%, 95% CI: 3.64, 4.81), *Trichuris trichiura* (2.51%, 95% CI: 2.17, 2.86), *Hymenolepis nana* (2.29%, 95% CI: 1.96, 2.63), *Taenia* species (1.01%, 95% CI: 0.80, 1.22), *Strongyloides stercoralis* (1.17%, 95% CI: 0.92, 1.41), and *Enterobius vermicularis*(0.71%, 95% CI: 0.52, 0.90) were recorded. Handwashing before food (OR: 5.22,95% CI: 3.49, 6.94), handwashing after toilet (OR: 3.03, 95%; CI: 1.01, 5.05), age (OR: 1.66, 95% CL. 1.09, 2.23), open defecation (OR: 2.42, 95% CI: 1.60, 3.24), eating raw and unwashed vegetables/fruits (OR: 1.98, 95%; CI: 1.30, 2.66), maternal education (OR: 1.81, 95% CI: 0.91, 2.72), family income (OR: 2.00, 95% CI: 0.87, 3.31), source of drinking water (OR: 3.12, 95% CI: 1.96, 4.27), swimming/contact with river water (OR: 1.90, 95% CI: 1.11, 2.69), barefoot (OR: 3.28, 95% CI: 1.67, 4.88), playing with soil (OR: 2.64, 95% CI: 1.40, 3.88), and family size (OR: 3.75, 95% CI: 2.03, 5.46) were factors associated with HIHPIs in Ethiopia. High heterogeneity of the prevalence of HIHPIs was observed among the studies within and among regions (I^2^ > 99.6% and *P* ≤ 0.001).

**Conclusion:**

HIHPIs in Ethiopia were significantly high. Therefore, special attention should be given by all stakeholders to minimize HIHPIs in Ethiopia.

## 1. Background

Parasitic infections caused by intestinal helminths are among the most prevalent global infections, especially in developing countries [1, 2]. The global annual burden of parasitic infections reaches 3.5 billion. Intestinal parasitic infections (IPIs) account for over 450 million annual morbidities and 200,000 mortalities [3]. Intestinal helminths (IHs) are among such infections that need special emphasis in developing countries [4]. They result in two billion global infections; Ascariasis (250 million), Schistosomiasis (200 million), hookworm (151 million), and Strongyloidiasis (100 million) [5]. Trichuriasis, another helminth disease, also infects around 800 million people worldwide [6].

The most frequent intestinal helminths in Ethiopia are *A. lumbricoides,* hookworm spp.*, S. mansoni, H. nana, T. trichiura, E. vermicularis, S. stercoralis,* and *Taenia* species [7]. Infections with these parasites usually lead to nutritional depletion, poor immunity in infants, mucosal loss and lymphatic leakage, and local hemorrhages [8]. Their associated factors are difficulties in adopting optimal personal hygienic practices, shoe-wearing habit, lack of clean and safe water, high population density, poor waste disposal, noncompliance with health standards, poor postdefecation handwashing, incorrect fingernail trimming, and eating raw meat/vegetables [1, 8–10]. Many studies have been conducted to determine the prevalence and associated risk factors of HIHPIs among people in different parts of Ethiopia. However, the prevalence reflected in these small and fragmented studies varied widely and remained inconclusive. Additionally, there is no nationwide study about the prevalence and factors associated with HIHPIs among the people in Ethiopia. Therefore, this review and meta-analysis aimed to determine the pooled prevalence and associated risk factors of HIHPIs among the people of Ethiopia.

## 2. Methods

### 2.1. Study Design and Setting

Ethiopia is located in the horn of Africa bounded by North and South Sudan on the west, Somalia and Djibouti on the East, Eritrea on the North and northwest, and Kenya on the South [11]. Ethiopian population is estimated to be 113,869,098. This population is equivalent to 1.47% of the global population. About 21.3% (24,463,423) of the Ethiopian population lives in urban areas. The country's population has a median age of 19.5 years. The population density of Ethiopia is 115 people km^−2^ (298 people mi^−2^). The total land area is 1,100,000 Km^2^ [12].

### 2.2. Search Strategies

We searched articles written in English on international databases such as Pub Med/MEDLINE, Science Direct, Web of Science, and Google Scholar [13]. Literature was collected within the time interval of December 2019-January 2020. The core search terms and phrases used were “prevalence,” “intestinal helminths parasites,” “associated risk factors,” and “Ethiopia”. The search terms were used separately and in combination with the Boolean operators “OR” or “AND”. Finally, the Preferred Reporting Items for Systematic Reviews and Meta-Analysis (PRISMA) checklist was used to present and report the results of the study [13].

### 2.3. Inclusion and Exclusion Criteria

Studies published from 2010 to January 2020 were included. All observational study designs (cross-sectional, case-control, and cohort) reporting the prevalence of intestinal parasitic infections and their associated risk factors among people in Ethiopia were included. Articles that were not accessible, articles written in languages other than English, and articles published before 2010 were excluded.

### 2.4. Outcomes of the Study

The measurement outcome of this study had two main outcome variables: the prevalence of HIHPIs and factors associated with HIHPIs. HIHPIs are defined as infections caused by one or more human helminth parasites [10].

### 2.5. Data Extraction

The data extraction protocol was prepared and evaluated by all authors We extracted information on the name of the author and year of publication, population studied, region and sites of study or focus, total sample size and the number of positives, estimated prevalence, species of intestinal parasites, and potential risk factors associated with individual species of HIHPs.

### 2.6. Quality Assessment of Individual Studies Included in the Meta-Analysis

The Grading of Recommendation Assessment Development and Evaluation (GRADE) approach was used to assess the overall quality of evidence [14]. Methodological quality, comparability of the outcome, and statistical analysis of the study were the three major assessment tools that were utilized to declare the quality of the study. Publications with total scores of five to six points were considered as high quality, three to four as moderate quality, and two and below as low quality.

### 2.7. Risk of Publication Bias across Studies Included in This Meta-Analysis

The risks of publication bias across the studies were assessed using funnel plot symmetry and Egger's test. The Egger's test *p*-value < 0.05 was used to determine the presence of publication bias across the studies. The cause of publication bias was assessed using a sensitivity test and regression test.

### 2.8. Data Analysis

The prevalence of HIHPIs was computed by dividing the total number of HIHPI cases by the total number of participants in the studies multiplied by 100. Besides, we used a random-effect model to estimate the pooled effect size (prevalence of HIHPIs). Cochrane Q-test and I^2^ statistics were used to assess heterogeneity among the studies [15]. To sort out the causes of heterogeneity, we conducted a subgroup analysis based on the region of the study, the nature of study participants, study year, and sample size in individual studies. Forest plot format was used to present the pooled point prevalence with 95% CI. A log odds ratio was used to decide the association between associated risk factors and the prevalence of HIHPIs among respondents. The meta-analysis was conducted using STATA Corp College Station, TX software version 14. *P*-values < 0.05 were considered statistically significant.

## 3. Result

**Figure 1:**
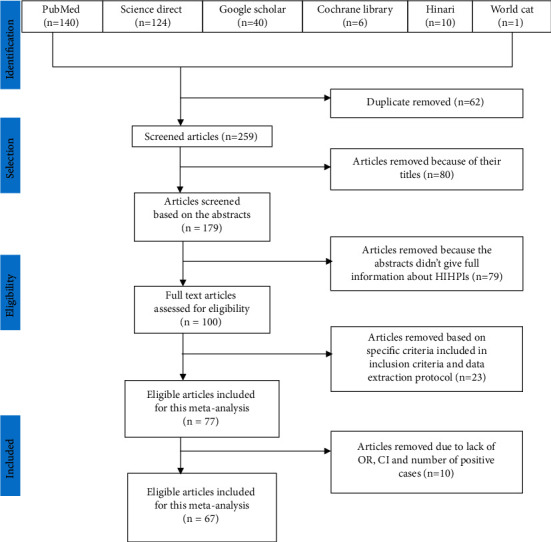
PRISMA flow diagram of articles considered for the review of HIHPIs among the Ethiopian population.

Out of the 321 articles retrieved, 62 articles were excluded due to duplicates, 80 articles due to their titles, and 79 due to their abstracts. The remaining 100 full-text articles were assessed for their eligibility. Twenty-three articles were excluded because of not fulfilling specific inclusion criteria and data extraction protocol. Consequently, 67 studies met the eligibility criteria and were included in the final meta-analysis (Figure 1).

### 3.1. Characteristics of Original Studies

Among the 67 studies selected, a total of 102, 265 study participants were involved to determine the pooled prevalence of HIHPIs and their associated risk factors among the people of Ethiopia. Among the studies, 85.07% and 14.9% were cross-sectional and retrospective studies, respectively. The sample size of the selected studies ranged from 118 to 21611 (Table 1).

Low prevalence of HIHPIs was reported in studies conducted in Woreta Health Center (2%) [50], Debre Berhan Hospital (3.2%) [71], among food handlers in Chagni town (4.8%) [23], among food handlers in AAU (5.8%) [18], St. Mary Hospital Axum (5.85%) [78], and inmates in Mekelle prison (5.8%) [44]. However, the highest prevalence was reported in a study conducted among street dwellers in Addis Ababa, Ethiopia (87.3%) [28].

Twenty-seven (40.3%) of the studies were from Amhara region, 13 (19.4%) from SNNP region, 15 (22.4%) from Oromia region, 3 (4.5%) from Addis Ababa city, 1 (1.5%) from Harare region, 1 (1.5%) from Benishangul-Gumuz region, and 7 (10.4%) from Tigray region (Table 1). However, no studies were reported from Afar, Dire Dawa, Gambella, and Somali regions.

### 3.2. Prevalence of Intestinal Helminth Parasitic Infections in Ethiopia

**Figure 2:**
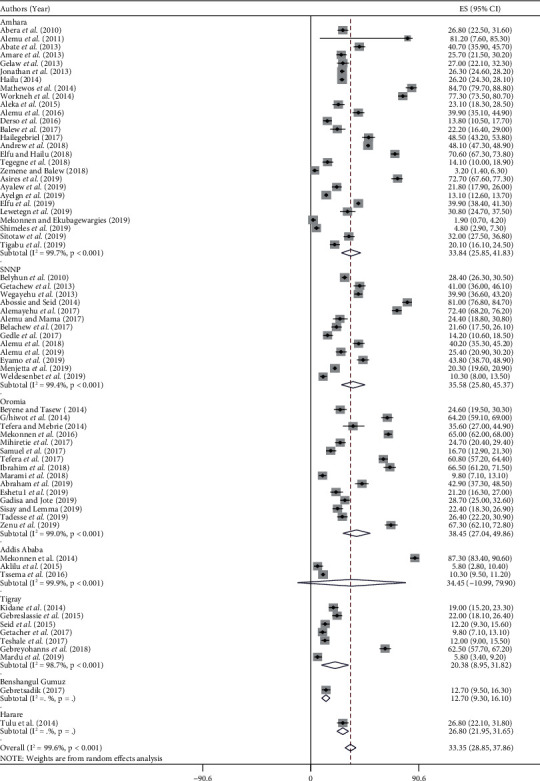
Forest plot showing the overall pooled prevalence of intestinal helminths in Ethiopia including the region level.

The overall national pooled prevalence of intestinal helminth parasitic infections in Ethiopia was 33.35% (95% CI; 28.85, 37.86). High heterogeneity was observed across the included studies (I^2^ = 99.6%, *P* ≤ 0.001). As a result, a random-effects model was employed to estimate the pooled prevalence of HIHPIs in Ethiopia (Figure 2).

### 3.3. Subgroup Analysis

**Figure 3:**
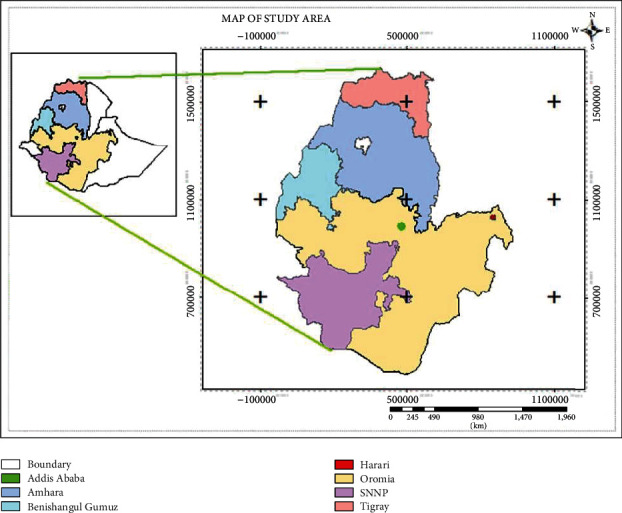
Regions from where the reports were obtained.

High pooled prevalence of HIHPIs was observed from Oromia region (38.45%, 95% CI: 27.04, 49.86), followed by SNNP region (35.58%, 95% CI: 25.80, 45.37), Addis Ababa city (34.45%, 95% CI: -10.99, 79.90), Amhara region (33.84%, 95% CI: 25.85, 41.83), Harare region (26.80%, 95% CI: 21.95, 31.65), and Tigray region (20.38%, 95% CI: 8.95, 31.82) in descending order (Figure 3). But, the lowest prevalence was observed from the Benishangul-Gumuz region (12.70%, 95% CI: 9.30, 16.10). Also, the pooled prevalence of HIHPIs was lower in studies having a sample size of (*n*) ≤ 300 (24.57%, 95% CI: 11.64, 37.49) as compared with those having sample size of (*n*) > 300, (35.27%, 95% CI: 30.34, 40.20) (Table 2).

Furthermore, the pooled prevalence of HIHPIs among studies conducted from 2010-2014 was 44.64% (95% CI: 34.39, 54.89) compared with studies conducted from 2015-2019 (29.08%, 95% CI: 24.12, 34.04). Based on the nature of the study subjects, the pooled prevalence of HIHPIs among people in Ethiopia was urban dwellers (53.45%, 95% CI: -2.28, 109.19), rural dwellers (51.76%, 95% CI: 38.14, 65.37), under-five children (37.83%, 95% CI: 26.19, 49.47), school children (36.33%, 95% CI: 28.51, 44.15), pregnant women (35.89%, 95% CI: 15.66, 56.12), food handlers (25.14%, 95% CI: 9.16, 41.11), and patients (17.96%, 95% CI 14.07, 21.85) in descending order (Table 2).

### 3.4. Common Intestinal Helminth Parasites among People in Ethiopia

The pooled prevalence of *A. lumbricoides* was (10.84%, 95% CI: 9.34, 12.34), followed by hookworm spp. (8.89%, 95% CI: 7.75, 10.04), *S. mansoni* (4.22%, 95% CI: 3.64, 4.81), *T. trichiura* (2.51%, 95% CI: 2.17, 2.86), *H. nana* (2.29%, 95% CI: 1.96, 2.63), *Taenia* species (1.01%, 95% CI: 0.80, 1.22), *S. stercoralis* (1.17%, 95% CI: 0.92, 1.41), and *E. vermicularis* (0.71%, 95% CI: 0.52, 0.90) among the people of Ethiopia (Table 3).

### 3.5. Risk of Publication Bias across the Studies Included in the Meta-Analysis

**Figure 4:**
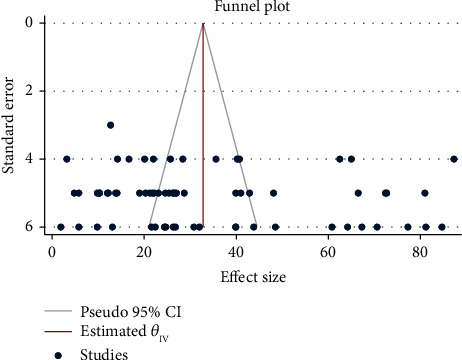
Funnel plot of the studies considered.

The funnel plot symmetry proves the presence of publication bias among the studies included in the present meta-analysis (Figure 4). Similarly, Egger's test results (*P* < 0.05) indicate the presence of a publication bias among the studies.

### 3.6. Factors Associated with HIHPIs among People in Ethiopia

Handwashing habits before food, handwashing after toilet, age, open field defecation, the habit of eating raw and unwashed vegetables/fruits, maternal education, levels of income, source of drinking water, swimming, walking on barefoot, playing with soil, and family size were significantly associated with HIHPIs.

Thirteen studies were used to test the association between HIHPIs and age among people of Ethiopia [5, 7, 8, 10, 21, 26, 29, 48, 50, 57, 60, 67, 77]. The pooled result of this meta-analysis indicated that age is significantly associated with the prevalence of HIHPIs. The odds of having HIHPIs in children up to 14 years was 1.66-fold higher than 14 years and older people (OR: 1.66 (95% CI. 1.09, 2.23) (Figure S1).

The association between family size and HIHPIs was computed in six studies [7, 10, 40, 48, 64]. People who had a family size above six were 3.75 times more likely to have HIHPIs than those who had a family size below six (OR: 3.75, 95% CI: 2.03, 5.46) (Figure S2). There was a significant association between family educational level and HIHPIs [10, 21, 25, 28, 31, 36, 40, 45, 57, 64]. Uneducated people were 1.81 times more likely to have HIHPIs than those who were educated (OR: 1.81, 95% CI: 0.91, 2.72) (Figure S3). The association between levels of income and HIHPIs was computed in six studies [19, 24, 27, 36, 59, 64]. People who had a low level of income were twice more likely to have HIHPIs than their counterparts (OR: 2.00, 95% CI: 0.87, 3.31) (Figure S4).

There was a significant association between the sources of drinking water and HIHPIs [7, 19, 25, 31, 40, 48, 58, 64, 69, 74]. People who drank untreated water were 3.21-folds more likely to have HIHPIs than those who drank treated water (OR: 3.12, 95% CI: 1.96, 4.27) (Figure S5). The association between handwashing before feeding and HIHIPs was computed from twenty studies [5, 7, 10, 19, 23–31, 45, 53, 59, 60, 64, 65, 69]. The odds of having HIHPIs among people who did not have hand washed habits before feeding was 5.22 times higher than those who had hand washed habits before feeding (OR: 5.22, 95% CI. 3.49, 6.94) (Figure S6).

Similarly, the association between handwashing after defecation with HIHPIs was evaluated using eight studies [7, 19, 26, 36, 41, 48, 53, 60]. The odds of having HIHPIs were 3.03 times higher among people who did not wash their hands after defecation than their counterparts (OR: 3.03, 95%; CI: 1.01, 5.05) (Figure S7). Furthermore, the association between open field defecation and intestinal helminth parasitic infection was computed in this meta-analysis [10, 25, 26, 29, 50, 56, 58, 60, 67, 79]. The odds of having HIHPIs among people who practiced open field defecation was 2.42 more likely to have HIHPIs than those who had not used open field defecation (OR: 2.42, 95% CI: 1.60, 3.24) (Figure S8).

The association between eating raw and unwashed vegetables/fruits with HIHPIs was evaluated in thirteen articles [10, 26, 28, 29, 31, 45–47, 50, 59–61, 79]. The odds of having HIHPIs among people who had the habit of eating raw and unwashed vegetables/fruits were 1.98 times higher than their counterparts (OR: 1.98, 95%; CI: 1.30, 2.66) (Figure S9).

Walking on barefoot was significantly associated with HIHPIs [7, 10, 19, 21, 29, 53, 61, 70]. People who had the habit of walking on barefoot were 3.28-fold more likely to have HIHPIs than their counterparts (OR: 3.28, 95% CI: 1.67, 4.88) (Figure S10). According to the meta-analysis of five studies [26, 50, 56, 57, 70], playing with soil was associated with HIHPIs. People who had the habit of playing with soil were 2.64 more likely to have HIHPIs than their counterparts (OR: 2.64, 95% CI: 1.40, 3.88) (Figure S11).

The results from the analysis of ten studies [7, 19, 21, 26, 33, 35, 47, 58, 61, 67] showed that swimming in the river was associated with HIHPIs among people in Ethiopia. People who had the habit of swimming in river waters were 1.90 times more likely to have HIHPIs than their counterparts (OR: 1.90, 95% CI: 1.11, 2.69) (Figure S12).

## 4. Discussion

Human intestinal helminth infections are among the major IPIs in Ethiopia and are the most common causes of morbidity [10]. The overall pooled prevalence of HIHPIs in this meta-analysis was (33.35%) among people in Ethiopia. It was higher than that of protozoa (25.01%) [80] and the global prevalence (24%) [81]. The outcome of this meta-analysis was higher than that of Brazil (10.1%) [82], Thailand (14.3%) [83], Uganda (26.5%) [84], Cameroon (28.6%) [85], and Cambodia (26.2%) [86]. However, it was in line with the study conducted in Tajikistan (32%) [87], and it was lower than the studies conducted in Lao (41.2%) [88] and Malaysia (50.4%) [89]. These differences could be due to methodological, socioeconomic, hygienic, sanitary, weather, climate, and environmental factors [90].

The prevalence of *A. lumbricoides* (10.84%) in this meta-analysis was lower than the global prevalence (15.5%) [91] and higher than the studies conducted in Co ˆte d'Ivoire (0.8%) [92], Tanzania (6.8%) [93], Cambodia (4.6%) [86], Brazil (5%) [82], and Western Tajikistan (4.4%) [87]. But, it was in line with the study conducted in Uganda (9.8%) [84]. However, it was lower than that from Malaysia (24.3%) [89], Rwanda (28.5%) [94], Cameroon (21.6%) [85], Sri Lanka (38.4%) [95], and Indonesia (53.5%) [96]. This difference might be due to differences in the eating habits of raw vegetables/fruits, environmental conditions [1], and socioeconomy status [97].

The prevalence of hookworm spp. (8.89%) in this meta-analysis was close to the global prevalence (10.1%) [98] and higher than the study conducted in Hawassa University students' clinic (2%) [63] and Brazil (1.0%) [82]. The outcome was in line with the study conducted in Bahir Dar, Ethiopia (6.2%) [7], and Cambodia (9.6%) [86]. However, it was lower than the study conducted in Uganda (18.5%) [84], Malaysia (22%) [89], and Indonesia (53.5%) [96]. This variation might be due to variation practices such as handwashing, disposal of waste, personal hygiene, and the wearing of shoes [99].

The prevalence of *S. mansoni* (4.22%) in this meta-analysis was higher than the global prevalence (3.1%) [100] and in studies conducted in Ghana (1.7%) [101], Gamo, Southern Ethiopia (0.12%) [8], and South Africa (0.9%) [102]. However, it was less than in Ethiopia (18.7%) [103]. This variation might be due to the difference in the distribution of helminth species in different geographical areas and method differences that might underestimate the detection of helminth infection [54]. Variations in the quality of water, irrigation activities and farming, swimming habits, and water contamination might be the other reasons associated with *S. mansoni* prevalence differences [104].

The prevalence of *T. trichiura* (2.51%) in this meta-analysis was lower than the global prevalence (10.3%) [105] and higher than the study conducted in Uganda (0.5%) [84] and Co ˆte d'Ivoire (1.2%) [92]. However, it was lower than the study conducted in Brazil (4.6%) [82], Malaysia (14.4%) [89], and Indonesia (60.4%) [96]. It might be because of the differences in toilet facilities, handwashing habits, and awareness of the transmission and prevention of helminth infections [66].

The prevalence of *H. nana* (2.29%) in this meta-analysis was lower than the global prevalence (4%) [106] and a study from South Africa (5.26%) [107]. However, it was higher than the studies conducted in Cambodia (0.2%) [86] and Ghana (0.3%) [101]. The possible reasons might be due to the residence, socioeconomic, sociodemographic, and environmental variations that favor fecal-oral transmission of the parasite [108].

The prevalence of the *Taenia* species (1.01%) in the present meta-analysis is close to the global prevalence (0.9%) [109] and is higher than in the study conducted in Cambodia (0.4%) [86]. However, it was lower than the study in South Africa (6.4%) [102]. This difference might be due to the variation in the environment and living conditions of the study participants [26]. Ethiopian people are known for eating raw meat [110].

The prevalence of *S. stercoralis* (1.17%) in this meta-analysis was higher than in the study conducted in Ghana (0.3%) [101]. However, it was lower than the study outcome of Angola (12.8%) [111] and Alabama (7.3%) [112]. Such variations in the prevalence of helminth infections attribute to variations in water supplies, sanitation, and hygiene [33]. Weakening immunity due to HIV/AIDS among HIV-positive people might also be the other reason for the variation.

The prevalence of *E. vermicularis* (0.71%) in this meta-analysis was in line with the study conducted in Tepi Town, South West Ethiopia (0.26%) [1, 9]. However, it was lower than the worldwide prevalence (2.3%) and the study conducted in Cambodia (1.1%) [86]. This variation might be due to the differences in the distribution of helminth species in different geographical areas, variations in water supplies, sanitation, hygiene, and methodology used during helminth identification [33, 54]. The habit of suckling fingers/learning materials and playing with soil may be the reasons for enterobiasis infections [113].

The subgroup analysis of this study indicated that the highest prevalence of HIHPIs was observed in Oromia region (38.45%, 95% CI: 27.04, 49.86), followed by SNNPR (35.58, 95% CI: 25.80, 45.37), Amhara region (33.84%, 95% CI: 25.85, 41.83), and Addis Ababa (34.45%, 95% CI: -10.99, 79.90). It was similar to the meta-analysis of SNNP (30.39%), Oromia (29.14%), and Amhara (27.55%) regions [114]. Whereas, low prevalence was observed from Harare (26.80%, 95% CI: 21.95, 31.65), Tigray (20.38%, 95% CI: 8.95, 31.82), and Benishangul-Gumuz (12.70%, 95% CI: 9.30, 16.10). The possible justification for this difference might be due to the peculiarities in sociodemographic, environmental, geographical, and behavioral characteristics. HIHPIs prevalence in five of the regions, namely, Oromia (38.45%), SNNPR (35.58%), Addis Ababa (34.45%), Amhara (33.84%), and Harare (26.80%) was higher than the global prevalence (24%) [81]. Prevalence of only the two regions: Tigray (20.38%) and Benishangul-Gumuz (12.70%) had a better prevalence compared to the globe.

The prevalence of HIHPIs in the first five years (2010-2014), 44.64% (95% CI: 34.39, 54.89), was higher than in the second round 2015-2019 (29.08%; 95% CI: 24.12, 34.04). The result showed that the trend of HIHPIs in Ethiopia was reduced. The outcome was similar to a systematic review, and meta-analysis was conducted in Ethiopia in which the pooled prevalence in the years 1997–2002, 2003–2008, 2009–2014, and >2014 was 71%, 42%, 48%, and 42%, respectively [115]. The potential reason for this decreasing rate might be due to the development of awareness about the transmission and prevention mechanisms of HIHPIs and mass deworming programs [116].

The prevalence of HIHPIs was the highest among urban dwellers (53.45%, 95% CI: −2.28, 109.19). It was higher than the report in the study conducted in the Gamo area, Ethiopia (39.9%) [66]. The lowest prevalence of HIHPIs was among patients (17.96%, 95% CI: 14.07, 21.85). It was similar to the study conducted in Shawura, Ethiopia (20.1%) [8]. These differences might come from the differences in diagnostic methods, population density, and geographical and behavioral characteristics.

The odds of having intestinal helminthic parasite infections in children up to the age of 14 years were 1.66 times higher than in adults. It agrees with the study conducted in tropical semiurban communities [117], Ghana [118], and Yemen [119]. Children are vulnerable to infections with HIHPIs because of a lack of well-developed immune systems and playing habits in fecal-contaminated soil [71].

People from large family sizes (greater than six) were 3.75 times more likely to have HIHPIs than those from family sizes less than six. It was similar to that of human intestinal protozoan parasitic infections (HIPPIs) (OR: 3.7, 95% CI: 1.45–5.85) [80]. It agrees with the studies performed in Ethiopia [40] and Ghana [101]. It might be because a large family size increases the chance of contact with each other and may also increase HIHPI transmission. On the other hand, large families could not get adequate medication and treatment.

Uneducated people were 1.81-fold more likely to have HIHPIs than the educated. This finding agreed with the study conducted in Kenya [120], Ghana [101], Bolivian [121], and South Africa [102]. It might be because uneducated people may lack the necessary knowledge and practice towards the transmission/prevention of intestinal helminth parasites.

People with a low-income level were twice likely to have HIHPIs compared to their counterparts. This finding agreed with the studies conducted in Dembiya district, North Ethiopia [19], and Haramaya University cafeterias [36]. It might be because low-income people fail to fulfill their sanitary requirements.

The odds of intestinal helminth parasite infections among people drinking untreated water were 3.12 times than those who used to drink treated water. It is in line with the studies from Indonesia [96], Uganda [122], and South Africa [102]. It might be because drinking and using untreated water may be a route for human helminth infections [123].

The odds of HIHPI occurrence among people who did not wash their hands before feeding were 5.22 times higher than those who did. This finding agrees with the studies conducted in Indonesia [96], the Eastern Region of Nepal [124], and Indonesia [125]. It might be because unwashed hands contain dust particles and microorganisms that facilitate the transmission of microorganisms fecal-orally.

Likewise, people who did not have handwashing habits after defecation were 3.03 times more likely to be infected with intestinal helminths than those who had handwashing habits. This result was higher than HIPPIs (OR: 2.82, 95% CI: 2.01-3.63) [80]. This finding agreed with the studies conducted in Indonesia [96], Uganda [122], Cameroon [125], and South Africa [102]. It might be because unwashed hands after defecation may contain stool materials and facilitate the transmission of microorganisms fecal-orally.

The odds of HIHPI occurrence were 2.42 times higher among different groups of people with open field defecation habits than their counterparts. It was close to that of human HIPPIs (OR: 2.91, 95% CI: 1.60–4.21) [80]. This finding agrees with a study conducted in Indonesia [96]. It might be because open field defecation would be a source of contamination to food and water sources.

People who had eaten leftover food and raw and unwashed vegetables/fruits were 1.98 times more likely to be infected with intestinal helminth parasites than those who had not. This finding is in line with the study conducted in Sri Lanka [95]. It might be because leftover food and raw and unwashed vegetables/fruits may contain HIHPs [123].

The odds of having intestinal helminth parasitic infections among people who had the habit of walking barefoot were 3.28 times higher than their counterparts. This finding agrees with studies conducted in Bolivia [121] and South Africa [102]. The reason might be helminths may have easy access into the body through skin penetration.

People who used to play with soil were 2.64 times more likely to be infected with intestinal helminth parasitic infections than those who did not. This finding agrees with the studies conducted in Turkey [126], Bolivia [121], and South Africa [102]. It might be because people who used to play with soil may have contact with soil-transmitted helminths' eggs, larvae, or adults [127].

Lastly, participants who used to swim in rivers were 1.90 times more likely to acquire HIHPIs than their counterparts. This finding is in line with the studies conducted in Ethiopia in Adi Remets town [21], Bahir Dar town [7], and Jimma town [61]. It might be because river waters may get contaminated with the ova, larvae, or adult helminth parasites.

## 5. Limitations of the Study

Lack of studies from Dire Dewa, Gambela, Somali, Afar, Harare, and Benishangul-Gumuz regions may underestimate both the pooled and subgroup prevalence of HIHPIs in Ethiopia. Lack of molecular techniques in the studies failed to identify hookworm species.

## 6. Conclusion

HIHPI prevalence among people in Ethiopia was high and still a major public health concern. The prevalent helminths identified were *Ascaris lumbricoides*, hookworm, *Schistosoma mansoni*, Trichuris trichiura, *Hymenolepis nana*, *Taenia* species, *Strongyloides stercoralis*, and *Enterobius* in descending order. Handwashing before food and after toilet, age, the habit of eating raw and unwashed vegetables/fruits, level of education, levels of income, source of drinking water, playing with soil, walking on barefoot, and family size were significantly associated with HIHPIs.

## 7. Recommendation

Particular emphasis shall be given to mass treatment and health education. Moreover, studies are needed in Gambella, Afar, Somali, Dire Dewa, Benishangul-Gumuz, and Harare regions.

## Figures and Tables

**Table 1:** Studies conducted in different parts of Ethiopia, their sample size, and prevalence.

	Region	Sample size	No. Positive	Prevalence (95% CI)	Quality score
Alemu et al. [5]	Amhara	319	263	81.2 (7.6,85.3)	6
Tigabu et al. [8]	Amhara	364	73	20.1(16.1,24.5)	6
Abate et al. [16]	Amhara	410	167	40.7 (35.9,45.7)	5
Derso et al. [17]	Amhara	384	53	13.8 (10.5, 17.7)	6
Aklilu et al. [18]	Addis Ababa	172	10	5.8 (2.8, 10.4)	6
Alemu et al. [19]	Amhara	401	160	39.9 (35.1, 44.9)	5
Fentahun et al. [7]	Amhara	418	91	21.8 (17.9, 26)	5
Tessema et al. [20]	Addis Ababa	4977	513	10.3 (9.5,11.2)	4
Gebreyohanns et al. [21]	Tigray	411	257	62.5 (57.7, 67.2)	6
Nute et al. [22]	Amhara	15455	7433	48.1(47.3, 48.9)	4
Alemu et al. [23]	Amhara	400	19	4.8 (2.9, 7.3)	6
Gelaw et al. [24]	Amhara	304	82	27 (22.1, 32.3)	5
Abossie and Seid [25]	SNNP	400	296	74 (76.8, 84.7)	5
Sisay and Lemma [26]	Oromia	384	86	21.9 (18.3, 26.9)	5
Asires et al. [27]	Amhara	344	250	72.7 (67.6, 77.3)	5
Mekonnen et al. [28]	Addis Ababa	355	310	87.3 (83.4, 90.6)	6
Sitotaw et al. [29]	Amhara	406	130	32 (27.5, 36.8)	6
Abera et al. [30]	Amhara	384	103	26.8 (22.5, 31.6)	4
Tulu et al. [31]	Harar	340	91	26.8 (22.1, 31.8)	6
Amare et al. [32]	Amhara	405	104	25.7 (21.5, 30.2)	6
Alemayehu et al. [33]	SNNP	503	364	72.3 (68.2, 76.2)	5
Feleke et al. [34]	Amhara	4436	1768	39.9 (38.4, 41.3)	6
Feleke and Jember [10]	Amhara	783	553	70.6 (67.3, 73.8)	6
Mathewos et al. [35]	Amhara	261	221	84.7 (79.7, 88.8)	5
Marami et al. [36]	Oromia	417	41	9.8 (7.1, 13.1)	6
Feleke et al. [37]	Tigray	21,611	1264	5.8 (7.1, 13.1)	6
Yesuf et al. [38]	Oromia	315	135	42.9 (37.3,48.5)	5
Gedle et al. [39]	SNNP	323	46	14.2 (10.6, 18.5)	5
Kidane et al. [40]	Tigray	384	73	19 (15.2, 23.3)	4
Tefera et al. [41]	Oromia	715	435	60.8 (57.2, 64.4)	5
Gadisa and Jote [42]	Oromia	561	161	28.7 (25, 32.6)	5
Belachew et al. [9]	SNNP	380	82	21.6 (17.5, 26.1)	5
Samuel et al. [43]	Oromia	317	53	16.7 (12.9, 21.3)	6
Mardu et al. [44]	Tigray	291	17	5.8 (3.4, 9.2)	6
Gebretsadik [45]	Gumuz	395	50	12.7 (9.5, 16.3)	4
Alemu and Mama [46]	SNNP	213	52	24 (18.8, 30.8)	3
Alemu et al. [47]	SNNP	391	157	40.2 (20.9, 30.2)	5
Alemu et al. [48]	SNNP	351	89	25.4 (35.3, 45.2)	6
Beyene and Tasew [49]	Oromia	260	64	24.6 (19.5, 30.3)	6
Mekonnen and Ekubagewargies [50]	Amhara	310	6	2 (0.7,4.2)	5
Weldesenbet et al. [51]	SNNP	600	62	10.3 (8, 13.5)	6
King et al. [52]	Amhara	2338	616	26.3 (20.4, 29.4)	4
Eshetu et al. [53]	Oromia	240	51	21.3 (24.6, 28.2)	4
Shiferaw et al. [54]	Amhara	180	40	22.2 (16.3, 27)	5
Ayelgn et al. [55]	Amhara	13329	1750	13.1 (16.4, 29)	5
Getachew et al. [56]	SNNP^*∗*^	388	159	41(12.6, 13.7)	5
Lewetegn et al. [57]	Amhara	214	66	30.8 (36, 46.1)	5
Seid et al. [58]	Tigray	442	54	12.2 (24.7, 37.5)	5
Gebrselassie et al. [59]	Tigray	404	89	22 (9.3, 15.6)	5
Tadesse et al. [60]	Oromia	417	110	26.4 (18.1, 26.4)	6
Zenu et al. [61]	Oromia	312	211	67.6 (22.2, 30.9)	4
Jember [62]	Amhara	2102	551	26.2 (62.1, 72.8)	6
Menjetta et al. [63]	SNNP^*∗*^	13679	2771	20.3 (24.3, 28.1)	4
Hailegebriel [64]	Amhara	359	174	48.5 (19.6, 20.9)	5
Tefera and Mebrie [65]	Oromia	118	42	35.6 (43.2, 53.8)	4
Wegayehu et al. [66]	SNNP^*∗*^	858	210	24.7 (27, 44.9)	5
Ibrahim et al. [67]	Oromia	340	226	66.5 (36.6, 43.2)	4
Addisu and Muche [68]	Amhara	365	134	36.7 (61.2, 71.5)	5
Eyamo et al. [69]	SNNP^*∗*^	384	168	43.8 (38.7, 48.9)	5
Workneh et al. [70]	Amhara	541	418	77.3 (73.5, 80.7)	5
Zemene and Shiferaw [71]	Amhara	247	8	3.2 (1.4, 6.3)	5
Teshale et al. [72]	Tigray	410	49	12 (9, 15.5)	4
Tegegne et al. [73]	Amhara	256	36	14.1(10,18.9)	6
Belyhun et al. [74]	Amhara	1813	515	28.4 (26.3, 30.5)	5
Aleka et al. [75]	Amhara	277	64	23.1 (18.3, 28.5)	6
Gebrehiwot et al. [76]	Oromia	374	240	64.2 (59.1, 69)	5
Mekonnen et al. [77]	Oromia	1021	664	65 (62, 68)	4

^*∗*^SNNP : Southern Nations, Nationalities, and Peoples Region.

**Table 2:** Pooled prevalence of intestinal parasite among People in Ethiopia, 2020 (*n* = 67).

Variable	Characteristic	Number of studies	Sample size	No. of positives	Prevalence (95% CI)	I-squared, *p*-value
Region	Oromia	15 (22.4%)	6163	2611	38.45% (27.04, 49.86)	99%, *p* ≤ 0.001
	SNNP	13 (19.4%)	20283	4971	35.58 (25.80, 45.37)	99.4%, *p* ≤ 0.001
	Addis Ababa	3 (4.5%)	5504	833	34.45 (−10.99, 79.90)	99.9%, *p* ≤ 0.001
	Amhara	27 (41.2%)	45627	15195	33.84 (25.85, 41.83)	99.7%, *p* ≤ 0.001
	Harare	1 (1.5%)	340	91	26.80 (21.95, 31.65)	I2 = , *p* =
	Tigray	7 (10.4%)	23953	1803	20.38 (8.95, 31.82)	98.7%, *p* ≤ 0.001
	Benishangul-Gumuz	1 (1.5%)	395	50	12.7 (9.5, 16.4)	I2 = , *p* =

Sample size	≤300	12	2729	671	24.57 (11.64, 37.49)	99%, *p* ≤ 0.001
	>300	55	99536	25043	35.27 (30.34, 40.20)	99.6%, *p* ≤ 0.001

Pooled prevalence among nature or study participants	School children	28	28228	11969	36.33(28.51, 44.15)	99.4%, *p* ≤ 0.001
	Patient	13	59638	7780	17.96 (14.07, 21.86)	99%, *p* ≤ 0.001
	Food handlers	7	2075	516	25.14 (9.16, 41.11)	99.2%, *p* ≤ 0.001
	Under five children	6	2218	848	37.83 (26.19, 49.47)	97%, *p* ≤ 0.001
	Pregnant women	6	2422	1032	35.89 (15.66, 56.12)	99.2%, *p* ≤ 0.001
	Rural dwellers	4	6726	3031	51.76 (38.14, 65.37)	98.9%, *p* ≤ 0.001
	Urban dwellers	3	958	538	53.45 (-2.28, 109.19)	99.8%, *p* ≤ 0.001

Pooled prevalence in years (trends)	2010-2014	19	12354	4681	44.64 (34.39, 54.89)	99.3%, *p* ≤ 0.001
	2015-2019	48	89911	21033	29.08 (24.12, 34.04)	99.6%, *p* ≤ 0.001

**Table 3:** Pooled prevalence of some common intestinal helminth parasites among people in Ethiopia.

Type of intestinal helminths parasite	No. of positivity	Pooled prevalence with 95% CI	I-squared
*A. lumbricoides*	9273	10.84 (9.34, 12.34)	99.2%, *p* ≤ 0.001
Hookworm	8471	8.89 (7.75, 10.04)	98.9%, *p* ≤ 0.001
*S. mansoni*	2979	4.22 (3.64, 4.81)	98.2%, *p* ≤ 0.001
*T. trichiura*	1887	2.51 (2.17, 2.86	96.9%, *p* ≤ 0.001
*H. nana*	1386	2.29 (1.96, 2.63),	91.7% *p* ≤ 0.001
*Taenia* species	619	1.01 (0.80, 1.22)	91.0%, *p* ≤ 0.001
*S. stercoralis*	598	1.17 (0.92, 1.41)	90.8%, *p* ≤ 0.001
*E. vermicularis*	381	0.71 (0.52, 0.90)	85.5%, *p* ≤ 0.001

## Data Availability

All related data have been presented within the article and supplementary data. The data set supporting the conclusions of this article is available from the corresponding author upon request.

## List of Abbreviations

AAU: Addis Ababa University
AIDS: Acquired immuno deficiency syndrome
CD4: Cluster of differentiation
HAART: Highly active antiretroviral therapy
HIHPIs: Human intestinal helminth parasitic infection
HIHPs: Human intestinal helminth parasites
HIPPIs: Human intestinal protozoan parasitic infections
HIV: Human immuno deficiency virus
IPIs: Intestinal parasitic infections
OR: Odds ratio
PRISMA: Preferred reporting items for systematic reviews and meta-analysis checklist
SNNP: Southern nations, nationalities, and people
STH: Soil-transmitted helminths
WHO: World Health Organization.

## Supplementary Materials

Supplementary Materials Figure S1. Odds ratio between age and HIHPIs in Ethiopia. Figure S2. Odds ratio between family size and HIHPIs in Ethiopia. Figure S3. Odds ratio between family education level and HIHPIs in Ethiopia. Figure S4. Odds ratio between income level and HIHPIs in Ethiopia. Figure S5. Odds ratio between source of drinking water and HIHPIs in Ethiopia. Figure S6. Odds ratio between no habit of handwashing before feeding and HIHPIs in Ethiopia. Figure S7. Odds ratio between no handwashing after defecation and HIHPIs in Ethiopia. Figure S8. Odds ratio between open-field defecation and HIHPIs in Ethiopia. Figure S9. Odds ratio between eating raw food and HIHPIs in Ethiopia. Figure S10. Odds ratio between walking on barefoot and HIHPIs in Ethiopia.
