# Supplementary material for: Prevalence and Associated Risk Factors of Human Intestinal Helminths Parasitic Infections in Ethiopia: A Systematic Review and Meta-Analysis
Source: ScientificWorldJournal. 2022 Aug 31;2022:3905963. doi: 10.1155/2022/3905963 (PMC9451958; doi:10.1155/2022/3905963)
Supplement: Supplementary Materials — Figure S1. Odds ratio between age and HIHPIs in Ethiopia. Figure S2. Odds ratio between family size and HIHPIs in Ethiopia. Figure S3. Odds ratio between family education level and HIHPIs in Ethiopia. Figure S4. Odds ratio between income level and HIHPIs in Ethiopia. Figure S5. Odds ratio between source of drinking water and HIHPIs in Ethiopia. Figure S6. Odds ratio between no habit of handwashing before feeding and HIHPIs in Ethiopia. Figure S7. Odds ratio between no handwashing after defecation and HIHPIs in Ethiopia. Figure S8. Odds ratio between open-field defecation and HIHPIs in Ethiopia. Figure S9. Odds ratio between eating raw food and HIHPIs in Ethiopia. Figure S10. Odds ratio between walking on barefoot and HIHPIs in Ethiopia. [file 3905963.f1.docx]

NOTE: Weights are from random effects analysis

Overall (I-squared = 75.0%, p < 0.001)

Sisay and Lemma *(*2019)

Sitotaw *et al.* (2019)

Alemu *et al.* (2011)

Mekonen and Ekubagewargies *(*2019)

Elfu and Hailu *(*2018)

Tadesse *et al.* (2019)

Alemu *et al.* (2019)

Mekonnen *et al.* (2016)

Tigabu *et al.* (2019)

Ibrahim *et al.* (2018)

Ayalew *et al.* (2019)

Lewetegn *et al.* (2019)

Gebreyohanns *et al.* (2018)

**Authors (Year)**

1.66 (1.09, 2.23)

0.31 (0.11, 0.86)

2.31 (1.14, 4.71)

3.08 (1.02, 9.37)

3.40 (1.27, 10.86)

6.48 (2.91, 14.40)

2.29 (1.33, 3.97)

2.51 (1.41, 4.45)

1.70 (1.20, 2.40)

0.64 (0.46, 0.90)

2.70 (1.20, 6.07)

**ES (95% CI)**

2.27 (1.41, 3.67)

1.85 (1.02, 3.34)

10.30 (1.48, 71.62)

0

-71.6

0

71.6

Figure S1. Odds ratio between Age and HIHPIs in Ethiopia

NOTE: Weights are from random effects analysis

Overall (I-squared = 82.7%, p < 0.001)

Elfu and Hailu (2018)

Kidane *et al.* (2014)

**Author (year)**

Alemu *(*2019)

Elfu *et al.* (2019)

Hailegebriel, (2017)

Ayalew *et al.* (2019)

3.75 (2.03, 5.46)

6.87 (3.67, 12.90)

13.64 (12.60, 22.40)

**ES (95% CI)**

1.50 (0.88, 2.55)

2.14 (1.60, 2.80)

4.90 (2.03, 11.83)

2.74 (1.25, 5.99)

0

-22.4

0

22.4

Figure S2. Odds ratio between family size and HIHPIs in Ethiopia

NOTE: Weights are from random effects analysis

Overall (I-squared = 76.2%, p < 0.001)

Abossie and Seid (2014)

Abossie and Seid *(*2014)

Tulu *et al.* (2014)

Gebreyohanns *et al.* (2018)

Mekonnen *et al.* (2014)

Hailegebriel *(*2017)

Elfu and Hailu (2018)

Marami *et al.* (2018)

Kidane *et al.* (2014)

**Author (Year)**

Lewetegn *et al.* (2019)

Gebretsadik *(*2017)

1.81 (0.91, 2.72)

2.98 (1.36, 6.53)

2.98 (1.36, 6.53)

0.27 (0.11, 0.67)

0.52 (0.07, 3.94)

1.20 (0.40, 3.60)

9.59 (3.26, 28.24)

2.32 (1.04, 5.26)

2.13 (1.24, 3.67)

2.51 (2.10, 4.02)

**ES (95% CI)**

2.40 (0.18, 5.60)

1.88 (1.12, 4.42)

0

-28.2

0

28.2

Figure S3. Odds ratio between family education level and HIHPIs in Ethiopia

NOTE: Weights are from random effects analysis

Overall (I-squared = 54.1%, p = 0.053)

Marami *et al.* (2018)

Gelaw *et al. (*2013)

Asires *et al.* (2019)

Hailegebriel (2017)

Alemu *et al. (*2016)

Author (Year)

GebreSlassie *et al. (*2015)

2.00 (0.87, 3.13)

3.86 (1.62, 9.20)

1.59 (0.26, 1.86)

0.75 (0.26, 2.16)

6.48 (2.42, 17.30)

8.90 (2.27, 25.40)

ES (95% CI)

3.09 (1.87, 5.09)

0

-25.4

0

25.4

Figure S4. Odds ratio between income level and HIHPIs in Ethiopia

NOTE: Weights are from random effects analysis

Overall (I-squared = 94.8%, p < 0.001)

Ayalew *et al.* (2019)

Eyamo *et al.* (2019)

**Author (Year)**

Alemu (2019)

Belyhun *et al.* (2010)

Ayalew *et al.* (2019)

Alemu *et al. (*2016)

Seid *et al.* (2015)

Workneh *et al.* (2014)

Kidane *et al.* (2014)

Abossie and Seid (2014)

Hailegebriel (2017)

Tulu *et al.* (2014)

Workneh *et al.* (2014)

3.11 (1.96, 4.26)

4.40 (2.32, 8.36)

0.10 (0.04, 0.28)

**ES (95% CI)**

1.58 (0.56, 4.49)

0.20 (0.06, 0.69)

4.40 (2.32, 8.36)

3.90 (1.20, 12.30)

2.70 (1.40, 55.20)

4.38 (3.14, 7.93)

8.97 (7.02, 9.60)

1.97 (0.43, 8.95)

2.84 (1.09, 7.45)

2.28 (1.19, 4.34)

4.38 (3.14, 7.93)

0

-55.2

0

55.2

Figure S5. Odds ratio between source of drinking water and HIHPIs in Ethiopia

NOTE: Weights are from random effects analysis

Overall (I-squared = 87.6%, p < 0.001)

Gebretsadik (2017)

Asires *et al.* (2019)

Abossie and Seid (2014)

Tulu *et al.* (2014)

**Author (Year**

Ayalew *et al.* (2019)

GebreSilassie *et al. (*2015)

Gelaw *et al. (*2013)

Elfu and Hailu (2018)

Tefera and Mebrie (2014)

Abera *et al. (*2010)

Sitotaw *et al.* (2019)

Tadesse *et al.* (2019)

Alemu *et al.* (2011)

Sisay and Lemma (2019)

Hailegebriel (2017)

Mekonnen *et al.* (2014)

Alemu *et al. (*2016)

Eshetu *et al.* (2019)

Eyamo *et al.* (2019)

Ayalew *et al.* (2019)

Shimeles *et al.* (2019)

5.22 (3.49, 6.94)

5.45 (4.55, 10.87)

7.33 (1.82, 10.87)

7.19 (0.95, 10.87)

0.20 (0.10, 10.87)

**ES (95% CI)**

2.48 (1.49, 10.87)

11.24 (6.73, 11.87)

6.45 (4.55, 11.90)

3.33 (1.54, 10.87)

7.80 (2.80, 24.80)

1.38 (0.83, 1.38)

5.00 (1.34, 10.87)

3.18 (1.53, 10.87)

0.09 (0.01, 10.87)

4.96 (1.77, 10.87)

2.33 (1.29, 10.87)

3.90 (0.20, 10.87)

6.45 (4.55, 11.90)

24.82 (19.38, 30.87)

3.41 (1.80, 6.46)

2.48 (1.49, 4.12)

4.77 (2.09, 10.87)

0

-30.9

0

30.9

Figure S6. The Odds ratio between no habit of handwashing before feeding and HIHPIs in Ethiopia

NOTE: Weights are from random effects analysis

Overall (I-squared = 82.2%, p < 0.001)

Tefera *et al.* (2017)

Marami *et al.* (2018)

Tadesse *et al.* (2019)

Alemu *et al.* (2016)

Alemu *et al.* (2019)

Sisay and Lemma (2019)

Ayalew *et al.* (2019)

**Author (Year)**

Eshetu *et al.* (2019)

3.03 (1.01, 5.05)

2.92 (1.09, 7.83)

2.43 (1.22, 4.86)

0.08 (0.03, 0.18)

7.30 (2.97, 17.95)

4.49 (2.00, 10.10)

3.39 (1.52, 7.57)

3.56 (2.07, 6.13)

**ES (95% CI)**

29.76 (21.90, 68.10)

0

-68.1

0

68.1

Figure S7. Odds ratio between no handwashing after defecation and HIHPIs in Ethiopia

NOTE: Weights are from random effects analysis

*Overall (*I-squared = 27.4%, p = 0.183)

Mekonen and Ekubagewargies *(*2019)

Tadesse *et al.* (2019)

Getachew *et al.* (2013)

Lewetegn *et al. (*2019)

Abossie and Seid *(*2014)

Seid *et al.* (2015)

Sitotaw *et al. (*2019)

**Authors (Year)**

Ibrahim *et al.* (2018)

Mihiretie *et al.* (2017)

Sisay and Lemma *(*2019)

Elfu and Hailu *(*2018)

2.42 (1.60, 3.24)

3.40 (1.27, 10.86)

6.45 (3.12, 13.32)

5.34 (1.99, 14.28)

1.85 (1.02, 3.34)

**ES (95% CI)**

3.62 (1.30, 10.04)

1.06 (0.45, 2.49)

2.33 (1.44, 3.76)

3.90 (1.41, 10.73)

4.62 (1.70, 8.30)

6.12 (2.41, 15.51)

2.94 (1.50, 5.80)

0

-15.5

0

15.5

Figure S8. Odds ratio between open-field defecation and HIHPIs in Ethiopia

NOTE: Weights are from random effects analysis

Overall (I-squared = 83.6%, p < 0.001)

Tadesse *et al.* (2019)

**Authors (Year)**

Mihiretie *et al.* (2017)

Zenu *et al.* (2019)

Sisay and Lemma *(*2019)

Alemu and Mama (2017)

Sisay and Lemma *(*2019)

Gebretsadik (2017)

Mekonnen *et al. (*2014)

Elfu and Hailu *(*2018)

Gebresilassie *et al. (*2015)

Sitotaw *et al.* (2019)

Mekonen and Ekubagewargies (2019)

Tulu *et al.* (2014)

Alemu *et al.* (2018)

1.98 (1.30, 2.66)

3.26 (1.55, 6.82)

3.30 (1.20, 6.30)

2.24 (1.04, 5.02)

2.80 (1.13, 6.96)

2.21 (1.03, 4.73)

**ES (95% CI)**

4.71 (1.63, 13.64)

0.31 (0.01, 0.63)

2.90 (1.02, 8.22)

2.08 (1.47, 2.94)

8.12 (2.16, 30.46)

1.82 (1.14, 2.30)

7.74 (1.61, 7.84)

0.46 (0.28, 0.75)

2.16 (1.10, 4.25)

0

-30.5

0

30.5

Figure S9. Odds ratio between eating raw food and HIHPIs in Ethiopia

NOTE: Weights are from random effects analysis

Overall (I-squared = 56.5%, p = 0.024)

Sitotaw *et al.* (2019)

Alemu *et al. (*2016)

Ayalew *et al.* (2019)

Zenu *et al.* (2019)

Gebreyohanns *et al.* (2018)

Eshetu *et al.* (2019)

**Author (Year)**

Elfu and Hailu (2018)

Workneh *et al.* (2014)

3.28 (1.67, 4.88)

4.00 (1.50, 9.44)

1.91 (1.01, 3.64)

4.19 (1.50, 11.65)

3.80 (1.80, 8.20)

8.13 (1.79, 36.90)

13.59 (12.81, 30.10)

**ES (95% CI)**

6.87 (3.67, 12.90)

1.38 (1.15, 3.10)

0

-36.9

0

36.9

Figure S10. Odds between walking on barefoot and HIHPIs in Ethiopia

NOTE: Weights are from random effects analysis

*Overall (*I-squared = 10.4%, p = 0.347)

**Authors (Year)**

Mekonen and Ekubagewargies (2019)

Workneh *et al.* (2014)

Getachew *et al.* (2013)

Lewetegn *et al.* (2019)

Sisay and Lemma (2019)

2.64 (1.40, 3.88)

2.43 (1.25, 5.18)

4.70 (2.00, 10.40)

4.64 (1.50, 14.36)

2.01 (1.04, 3.83)

8.57 (3.88, 18.91)

**ES (95% CI)**

0

-18.9

0

18.9

Figure S11. Odds ratio between playing with soil and HIHPIs in Ethiopia

NOTE: Weights are from random effects analysis

Overall (I-squared = 76.0%, p < 0.001)

Ayalew *et al.* (2019)

Ibrahim *et al.* (2018)

Alemu *et al.* (2016)

Alemayehu *et al.* (2017)

Zenu *et al.* (2019)

**Author (Year)**

Sisay and Lemma *(*2019)

Gebreyohanns *et al.* (2018)

Seid *et al.* (2015)

Mathewos *et al.* (2014)

Alemu *et al.* (2018)

1.90 (1.11, 2.69)

0.41 (0.21, 0.79)

4.89 (1.88, 8.65)

1.01 (0.08, 12.30)

2.95 (1.96, 4.45)

2.50 (1.24, 5.04)

**ES (95% CI)**

3.03 (1.12, 8.21)

2.06 (1.10, 5.73)

2.14 (1.09, 4.20)

2.54 (1.12, 5.74)

1.17 (1.03, 1.95)

0

-12.3

0

12.3

Figure S12. Odds ratio between swimming in river water and HIHPIs in Ethiopia
